# Activation of lateral preoptic neurons is associated with nest-building in male mice

**DOI:** 10.1038/s41598-024-59061-z

**Published:** 2024-04-09

**Authors:** Natsuki Tagawa, Keita Mori, Michinori Koebis, Atsu Aiba, Yuichi Iino, Yousuke Tsuneoka, Hiromasa Funato

**Affiliations:** 1 Department of Anatomy, Graduate School of Medicine, Toho University, Tokyo, 143-8540 Japan; 2 Department of Biological Sciences, Graduate School of Science, The University of Tokyo, Bunkyo-Ku, Tokyo, 113-0033 Japan; 3 Laboratory of Animal Resources, Center for Disease Biology and Integrative Medicine, Graduate School of Medicine, The University of Tokyo, Tokyo, 113-0033 Japan; 4 International Institute for Integrative Sleep Medicine (IIIS), University of Tsukuba, Tsukuba, Japan

**Keywords:** Neuroscience, Physiology

## Abstract

Nest-building behavior is a widely observed innate behavior. A nest provides animals with a secure environment for parenting, sleep, feeding, reproduction, and temperature maintenance. Since animal infants spend their time in a nest, nest-building behavior has been generally studied as parental behaviors, and the medial preoptic area (MPOA) neurons are known to be involved in parental nest-building. However, nest-building of singly housed male mice has been less examined. Here we show that male mice spent longer time in nest-building at the early to middle dark phase and at the end of the dark phase. These two periods are followed by sleep-rich periods. When a nest was removed and fresh nest material was introduced, both male and female mice built nests at Zeitgeber time (ZT) 6, but not at ZT12. Using Fos-immunostaining combined with double in situ hybridization of *Vgat* and *Vglut2*, we found that *Vgat*- and *Vglut2*-positive cells of the lateral preoptic area (LPOA) were the only hypothalamic neuron population that exhibited a greater number of activated cells in response to fresh nest material at ZT6, compared to being naturally awake at ZT12. Fos-positive LPOA neurons were negative for *estrogen receptor 1* (*Esr1*). Both *Vgat*-positive and *Vglut2*-positive neurons in both the LPOA and MPOA were activated at pup retrieval by male mice. Our findings suggest the possibility that GABAergic and glutamatergic neurons in the LPOA are associated with nest-building behavior in male mice.

## Introduction

Nest-building behavior is a widely observed innate behavior among various animal species^1–5^. A nest provides animals with a secure environment for sleep, feeding, reproduction, and parenting. For example, while asleep, animals render less protection from predators and decrease body temperature, during which a nest provides protection and has a heat retention function^6^. Since infants of nest-building animals generally spend their time in a nest, nest-building behavior is often regarded as one of the parental behaviors^2,7–9^. Reflecting the multifaceted purposes of a nest, nest-building behaviors have recently been variously examined as thermoregulatory behavior related to warm-sensitive neurons^10^, sleep preparatory behavior^11^, and maternal preparatory behavior^12^. Additionally, nest-building behavior is thought to be a circadian behavior that animals express at a specific time of day. However, the diurnal changes in nest-building behavior have not been examined in detail.

Several neurons have been reported to be involved in the regulation of nest-building behavior in a certain context and aim. The Edinger-Westphal nucleus and pontine formation were identified as the brain regions with increased Fos immunoreactivity in pregnant females compared to non-pregnant females and males in response to exposure to fresh nest material exposure and subsequent nest-building^12^. The excitotoxic lesion in the central and ventral regions of the preoptic area (POA) suppressed nest-building in female mice rearing pups^7^. Similarly, the optogenetic stimulation of the agouti-related neuropeptide (Agrp) neurons projecting to the medial preoptic area (MPOA) inhibited maternal nest-building^13^. Activation of warm-sensitive BDNF/PACAP-positive neurons in the ventromedial preoptic area suppressed nest-building as a cold-protective behavior under the lower ambient temperature^10^. The lateral hypothalamus is the only brain region that showed increased activated neurons using TRAP2 mice, specific to the nest-building group compared to sleep or sleep-deprived groups^11^. Thus, little research has been focused to identify brain regions for nest-building as a general innate behavior not related to parenting or cold challenge.

Through the course of our research on the functional and neuroanatomical analysis of the POA, we have revealed the cytoarchitecture of the POA according to gene expression profile and demonstrated the role of POA subregions in the regulation of various innate behaviors including nest-building^7–9,14^. The MPOA includes neurons that regulate sleep, parenting, body temperature regulation, sexual behaviors, and aggression^9^, whereas the lateral preoptic area (LPOA) includes neurons that regulate reward behaviors and process nociceptive stimuli^15,16^. Similar to these functional differences between MPOA and LPOA, the gene expression pattern also distinct between these areas. The galanin, neurotensin, and estrogen receptor are rich in the MPOA but devoid in the LPOA^9^.

In this study, we first investigated diurnal variation in nest-building of singly housed male mice and found that the motivation for nest-building varies with the time of day. We identified the LPOA as the hypothalamic region where the number of c-Fos-positive cells increased in response to fresh nest material and subsequent nest-building, compared to parenting and naturally awake mice. We further classified POA neurons activated by fresh nest material into inhibitory and excitatory neurons.

## Results

### Diurnal variation in nest-building of individually housed male mice

**Figure 1:**
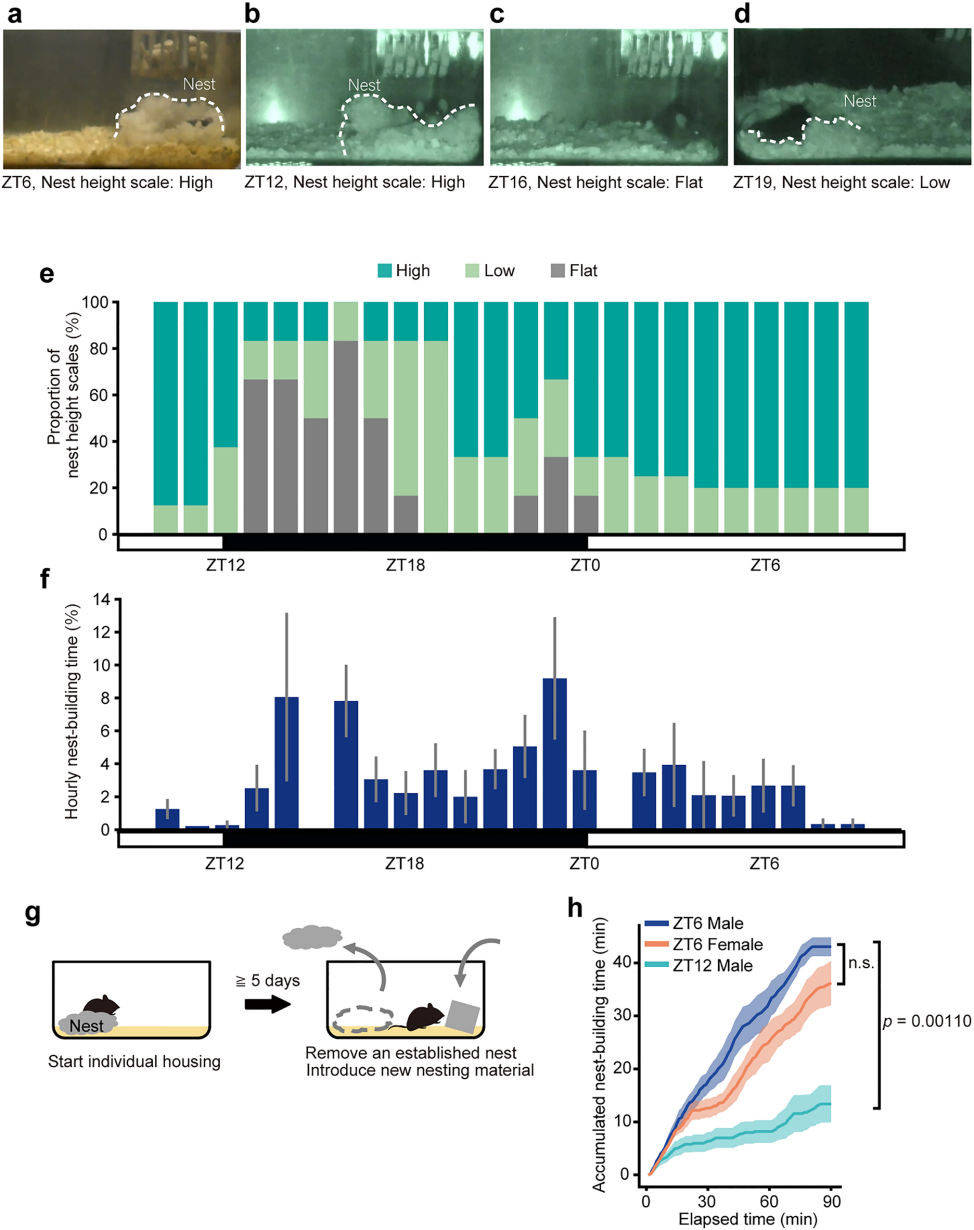
Diurnal variation of nest-building behavior and nest-building assay. (**a**–**d**) Representative pictures of nests at ZT6 (**a**), ZT12 (**b**), ZT16 (**c**), and ZT19 (**d**). (**e**) The proportion of nest height scales that was evaluated at that point in time every hour (ZT10-12, 8 mice; ZT13-1, 6 mice; ZT2-3, 8 mice; ZT4-9, 5 mice). (**f**) Relative time spent in nest-building (ZT10-12, 8 mice; ZT12-20, 6 mice; ZT20-22, 5 mice; ZT22-2, 6 mice; ZT2-3, 8 mice; ZT3-5, 4 mice and ZT5-10, 5 mice). (**g**) Diagram of the nest-building assay. After single-housed for five days or longer, the nest was removed and fresh nest material was immediately introduced to observe nest-building behavior. (**h**) Accumulated time manipulating nest material from the point of introducing the fresh nest material (ZT6-male, n = 5; ZT6-female, n = 5; ZT12-male, n = 5). Data are shown as the mean ± s.e.m.

First, we observed nest-building behavior of male mice that were individually housed with nest material from the side. Typically, mice slept in a high nest from the light phase (Fig. 1a) to the beginning of the dark phase (Fig. 1b), and, during the early dark phase, mice moved around in the cage and destroyed their nests flat or low (Fig. 1c,d). Subsequently, mice built nests again during the late dark phase. When rebuilding a nest from a scattered, flattened nest, mice engage in a certain set of behaviors (Supplementary Video): The mice collected and piled up nest materials scattered around the original nest position. Alternatively, from the exterior of the nest, the mice piled up the dispersed nest materials by pushing them toward the interior of the nest. The mice adjusted the shape and height of the nest from within the nest by manipulating and pushing nest materials with their forelegs and mouths. Occasionally, mice burrowed under the nests that were in the process of being built. Thus, evaluating nest height is an appropriate indicator of nest quality and nest-building behavior.

To examine the diurnal variation in nest-building, we evaluated the nest height of singly housed male mice in their home cages every hour from ZT10 for 24 h (Fig. 1e and Supplementary Fig. S1). At ZT12, the beginning of the dark phase, five of the eight nests were high (Low: 3/8 mice; High: 5/8 mice). Soon after the onset of the dark phase, the nests were destroyed, and five of the six nests were flat at ZT16 (Flat: 5/6 mice; Low: 1/6 mice), showing the highest proportion of flat nests throughout the day. The proportion of flat nests decreased and that of high nests increased toward ZT20 and 21. Flat nests reappeared at ZT22 and showed a small peak (2/6 mice) at ZT23. No flat nest was observed after ZT1 during the light phase. The proportion of high nests remained high (4 of 5 mice) during the mid to late light phase.

At the same time, we further investigated the time spent in building nests. At ZT12, whereas mice are most active, mice showed nest-building behavior for only very short time (0.3 ± 0.3%, n = 6). During the early dark phase, the time spent in building nests tended to be short with a large individual difference. Nest-building time was short during the mid dark phase. Around ZT20, nest-building time was shortest during the dark phase. Conversely, at the end of the dark phase, time spent in nest-building was the longest of the day. Nest-building time was short during the light phase (Fig. 1f). We also conducted a statistical analysis of the diurnal variation in time spent in building nests using general liner model. The analysis revealed a significant time-of-day effect in the time spent in building nests (*F*_23,108_ = 1.93, *p* = 0.0131). Of each hour of the day, estimated coefficients for nest-building behavior significantly increased at ZT23 (5.58 ± 2.56%, Wald *t* = 2.181, *p* = 0.0314).

We then compared the inactive time ratio for the pre-nest-building and post-nest-building during the dark and light phases (Supplementary Fig. S2). Inactive time ratio was higher in post nest-building than in pre nest-building in both the dark and the light phases (the dark phase, p < 0.001; the light phase, p = 0.0374). This result suggests that nest-building in a single-housed mice may be a preparatory behavior for resting and sleeping.

### Robust motivation for nest-building in males at ZT6

To examine whether the motivation for nest-building varies by time of the day, we conducted a nest-building assay (Fig. 1f), in which old nests were removed and fresh nest material was immediately introduced in the home cage. The time spent in handling or biting nest material during the 90 min-period was obtained. Mice spent most of their time sleeping at ZT6 when not disturbed, but fresh nest material kept them awake to build nests (Supplementary Fig. S3). Male mice spent a longer time in nest-building when given fresh nest material at ZT6 than at ZT12 (Fig. 1g) (43.0 ± 1.8 min for ZT6, 13.4 ± 3.5 min for ZT12, *p* = 0.00110). Ninety minutes after providing fresh nest material, all males in the ZT6 group built a high nest (5 mice). In contrast, no males in the ZT12 group built a nest (5 mice), regardless of whether they were awake (3 mice) or asleep (2 mice) at ZT12. Thus, at ZT12, male mice did not spontaneously build nests or nest-building in response to fresh nest material. In contrast, at ZT6, male mice did not spontaneously build nests, but they did build nests in response to fresh nest material. Additionally, there was no significant difference in time spent in nest-building at ZT6 between males and females (43.0 ± 1.8 min for males, 36.2 ± 4.2 min for females, *p* > 0.05).

### Fresh nest material activated specific hypothalamic areas

**Figure 2:**
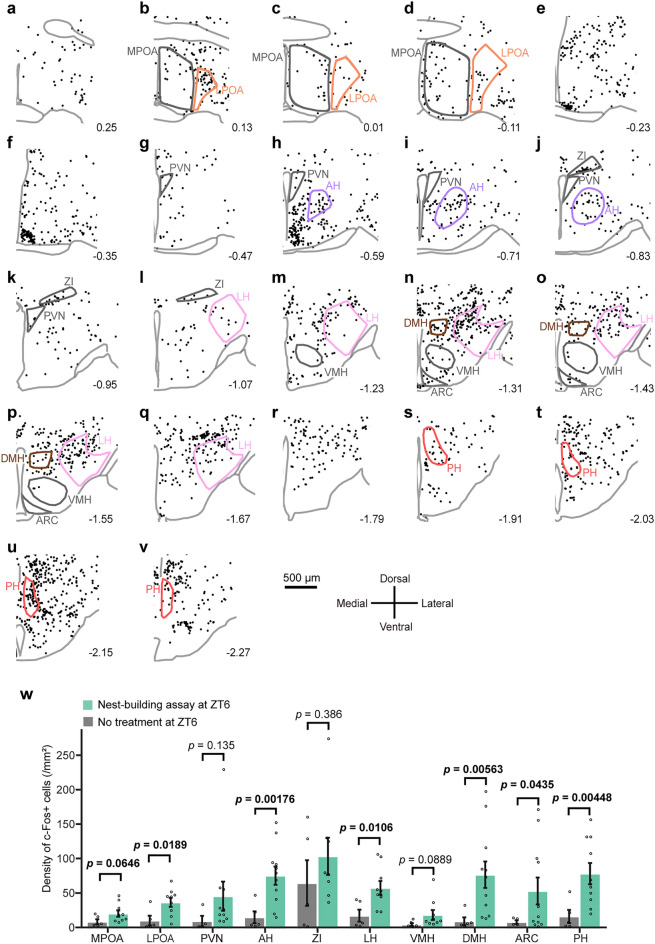
Nest-building activated multiple hypothalamic regions in males. (**a**–**v**) Coronal brain sections show the distribution of c-Fos-immunoreactive cells induced by nest-building in the hypothalamus. Each dot indicates a c-Fos-immunoreactive cell. The digit indicates the distance from the bregma. Scale bar 500 μm. Enclosed areas were assessed for the density of c-Fos + cells for (**w**). (**w**) Density of c-Fos-immunoreactive cells of nest-building assay at ZT6 (MPOA, n = 9; LPOA, n = 9; PVN, n = 10; AH, n = 11; ZI, n = 8; LH, n = 9; VMH, n = 9; DMH, n = 11; ARC, n = 11; PH, n = 10) and of ad libitum at ZT6 (MPOA, n = 5; LPOA, n = 5; PVN, n = 4; AH, n = 5; ZI, n = 5; LH, n = 5; VMH, n = 5; DMH, n = 5; ARC, n = 4; PH, n = 5). Welch’s t-test. Data are shown as the mean ± s.e.m.

Since the hypothalamus is involved in the regulation of behaviors closely related to nest-building, such as warm-sensing, sleep/wakefulness, and parenting^8–11,17^, we examined which hypothalamic areas have more neurons that are activated in response to fresh nest material. We harvested brains 120 min after providing fresh nest material to singly housed male mice at ZT6 and performed c-Fos immunostaining. Although c-Fos-positive cells were detected throughout the hypothalamus, the density of c-Fos-positive cells varied widely (Fig. 2a–v). When the density of c-Fos-positive cells in each hypothalamic area was calculated and compared with that of mice with no treatment at ZT6 (Fig. 2w), a significant increase in c-Fos positive cell density was recognized in the LPOA, anterior hypothalamic area (AH), lateral hypothalamic area (LH), dorsomedial hypothalamic nucleus (DMH), arcuate nucleus (ARC), and posterior hypothalamus (PH) (LPOA, *p* = 0.0189; AH, *p* = 0.00176; LH, *p* = 0.0106; DMH, *p* = 0.00563; ARC, *p* = 0.0435; PH, *p* = 0.00448). On the other hand, the density of c-Fos-positive cells in the MPOA, paraventricular hypothalamic nucleus (PVN), zona incerta (ZI), and ventromedial hypothalamic nucleus (VMH) did not show significant changes (*p* > 0.05).

### Fresh nest material activated GABAergic and glutamatergic neurons in the LPOA

**Figure 3:**
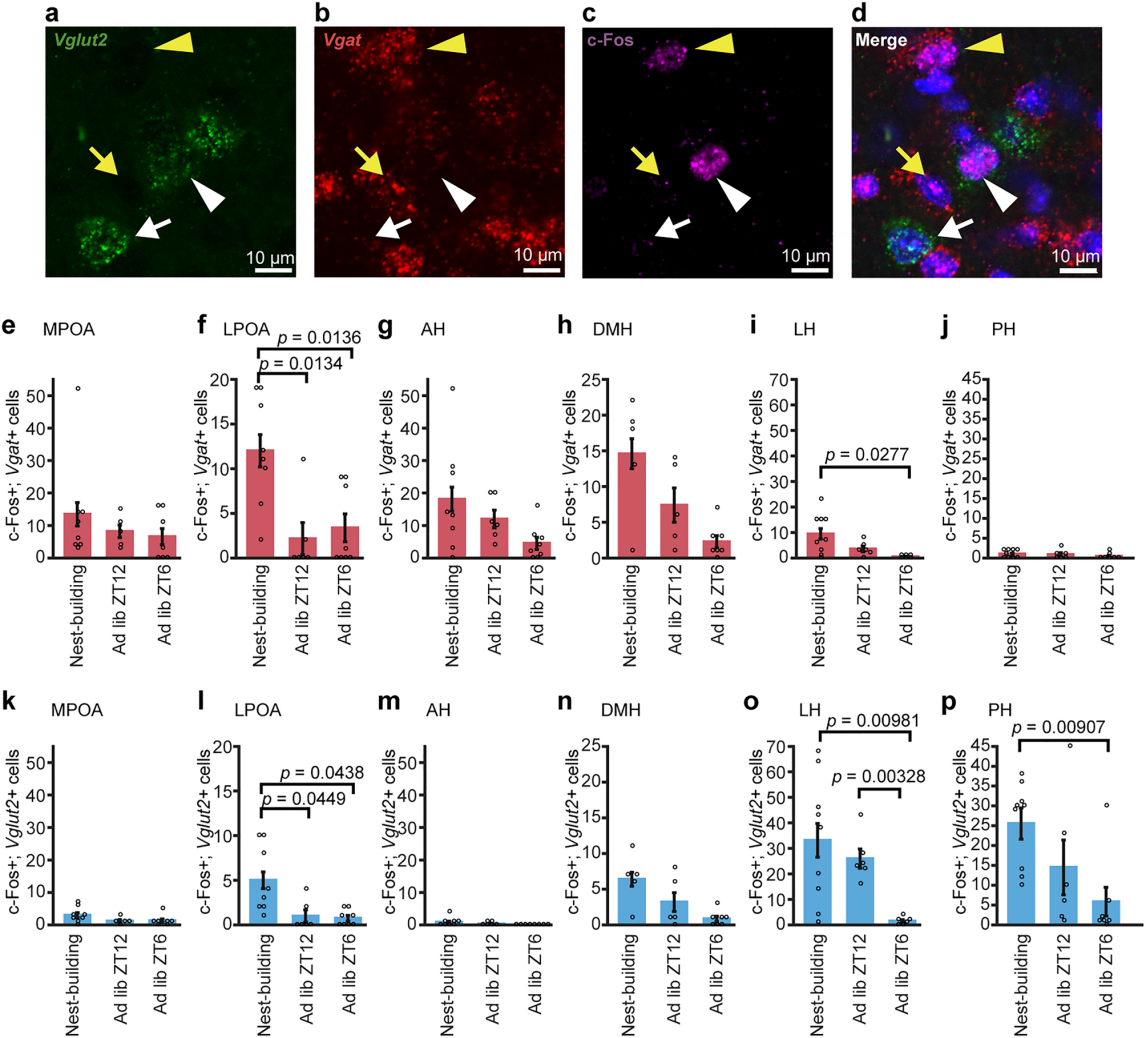
Excitatory and inhibitory populations of c-Fos-positive hypothalamic neurons associated with nest-building in males. (**a**–**d**) Representative microscopic images of c-Fos immunostaining with double in situ hybridization for Vglut2 and Vgat mRNA. A white arrowhead indicates a c-Fos- and Vglut2-positive cell and a yellow arrowhead indicates a c-Fos–Vgat-positive cell. A white arrow indicates a Vglut2-positive cell and a yellow arrow indicates a Vgat-positive cell. Scale bars: 10 μm. (**e**–**j**) The number of c-Fos and Vgat double-positive cells in the MPOA (**e**), LPOA (**f**), AH (**g**), DMH (**h**), LH (**i**), and PH (**j**) in the nest-building group, ad lib ZT12 group, and ad lib ZT6 group. (**k**–**p**) The number of c-Fos and Vglut2 double-positive cells in the nest-building group, ad lib ZT12 group, and ad lib ZT6 group in the MPOA (**k**), LPOA (**l**), AH (**m**), DMH (**n**), LH (**o**), and PH (**p**) (MPOA: nest-building, n = 8; ad lib ZT12, n = 5; ad lib ZT6, n = 7. LPOA: nest-building, n = 8; ad lib ZT12, n = 6; ad lib ZT6, n = 8. AH: nest-building, n = 8; ad lib ZT12, n = 6; ad lib ZT6, n = 8. DMH: nest-building, n = 5; ad lib ZT12, n = 5; ad lib ZT6, n = 7. LH: nest-building, n = 9; ad lib ZT12, n = 6; ad lib ZT6, n = 6. PH: nest-building, n = 9; ad lib ZT12, n = 6; ad lib ZT6, n = 7). Each p-value was adjusted by Holm's method after Welch's t-test. Data are shown as the mean ± s.e.m.

Given that neurons activated in response to fresh nest material include neurons associated with arousal state rather than nest-building behavior and that inhibitory and excitatory neurons may play different roles in regulating nest-building, we performed c-Fos immunostaining combined with double in situ hybridization for *Vgat* and *Vglut2* (Fig. 3a–d) in mice exposed to fresh nest material at ZT6 (nest-building group), mice housed with nest material spontaneously awake at ZT12 under ad libitum condition (ad lib ZT12 group), and mice housed with nest material slept at ZT6 under ad libitum condition (ad lib ZT6 group).

In the MPOA, there was no significant difference in c-Fos-positive cell number between groups neither in *Vgat*-positive or *Vglut2*-positive neurons (Fig. 3e,k). Among c-Fos-positive cells, the proportion of *Vgat*-positive cells was similarly greater than that of *Vglut2*-positive cells among the nest-building, ad lib ZT12, and ad lib ZT6 groups (Table 1).

**Table 1:** The proportion of *Vgat-*positive cells or *Vglu*t-positive cells among c-Fos positive cells in each group of virgin males.

Virgin males			
	Nest-building	Ad lib ZT12	Ad lib ZT6
MPOA			
Vgat	70.6%	71.9%	76.7%
Vglut2	15.7%	10.5%	15.0%
LPOA			
Vgat	66.2%	56.5%	73.0%
Vglut2	27.6%	26.1%	16.2%
AH			
Vgat	94.8%	94.7%	94.7%
Vglut2	4.6%	2.6%	0.0%
DMH			
Vgat	59.3%	56.9%	55.2%
Vglut2	26.0%	24.6%	20.7%
LH			
Vgat	18.9%	9.9%	20.0%
Vglut2	66.2%	73.2%	60.0%
PH			
Vgat	3.2%	4.6%	6.1%
Vglut2	81.0%	80.6%	83.7%

In the LPOA, the number of c-Fos- and *Vgat*-positive cells of the nest-building group was significantly higher than that of other groups (nest-building vs. ad lib ZT6, *p* = 0.0136; nest-building vs. ad lib ZT12, *p* = 0.0134) (Fig. 3f). The number of c-Fos- and *Vglut2*-positive cells of the nest-building group was also significantly higher than that of other groups (nest-building vs. ad lib ZT6, *p* = 0.0438; nest-building vs. ad lib ZT12, *p* = 0.0449) (Fig. 3l). Among c-Fos-positive cells, the percentage of *Vgat*-positive cells was similarly greater than that of *Vglut2*-positive cells among the nest-building, ad lib ZT12, and ad lib ZT6 groups (Table 1). In the MPOA and LPOA, there was no significant correlation between the number of c-Fos-positive cells and the age of the mice (Supplementary Fig. S4).

In the AH and DMH, there was no difference in the number of c-Fos-positive cells between groups neither in *Vgat*-positive or *Vglut2*-positive cells (Fig. 3g,h,m,n). Among c-Fos-positive cells, the proportion of *Vgat*-positive cells was greater than that of *Vglut2*-positive cells in the nest-building, ad lib ZT12, and ad lib ZT6 groups in the AH and, the nest-building, ad lib ZT12, and ad lib ZT6 groups in the DMH (Table 1).

In the LH, the number of c-Fos- and *Vgat*-positive cells of the nest-building group was higher than that of the ad lib ZT6 group (*p* = 0.0277) but was not significantly different from that of the ad lib ZT12 group (Fig. 3i). The numbers of c-Fos- and *Vglut2*- positive cells of the nest-building group and the at lib ZT12 group were higher than that of the ad lib ZT6 group (nest-building vs. ad lib ZT6, *p* = 0.00981; ad lib ZT12 vs. ad lib ZT6, *p* = 0.00328) but was not significantly different from that of the ad lib ZT12 group (Fig. 3o). Among c-Fos-positive cells, the proportion of *Vglut2*- positive cells was greater than that of *Vgat*-positive cells in the nest-building, ad lib ZT12, and ad lib ZT6 groups (Table 1).

In the PH, the number of c-Fos- and *Vgat*-positive cells of the nest-building group was not significantly different from that of the other groups (Fig. 3j), whereas the number of c-Fos- and *Vglut2*-positive cells of the nest-building group was higher than that of the ad lib ZT6 group (nest-building vs. ad lib ZT6, *p* = 0.00907) and not significantly different from that of the ad lib ZT12 group (Fig. 3p). Among c-Fos-positive cells, the proportion of *Vglut2*-positive cells was similarly greater than that of *Vgat*-positive cells among the nest-building, ad lib ZT12, and ad lib ZT6 groups (Table 1).

These results suggest that GABAergic and glutamatergic neurons in the LPOA may include neurons that are specifically activated in fresh nest material and enhance nest-building.

**Figure 4:**
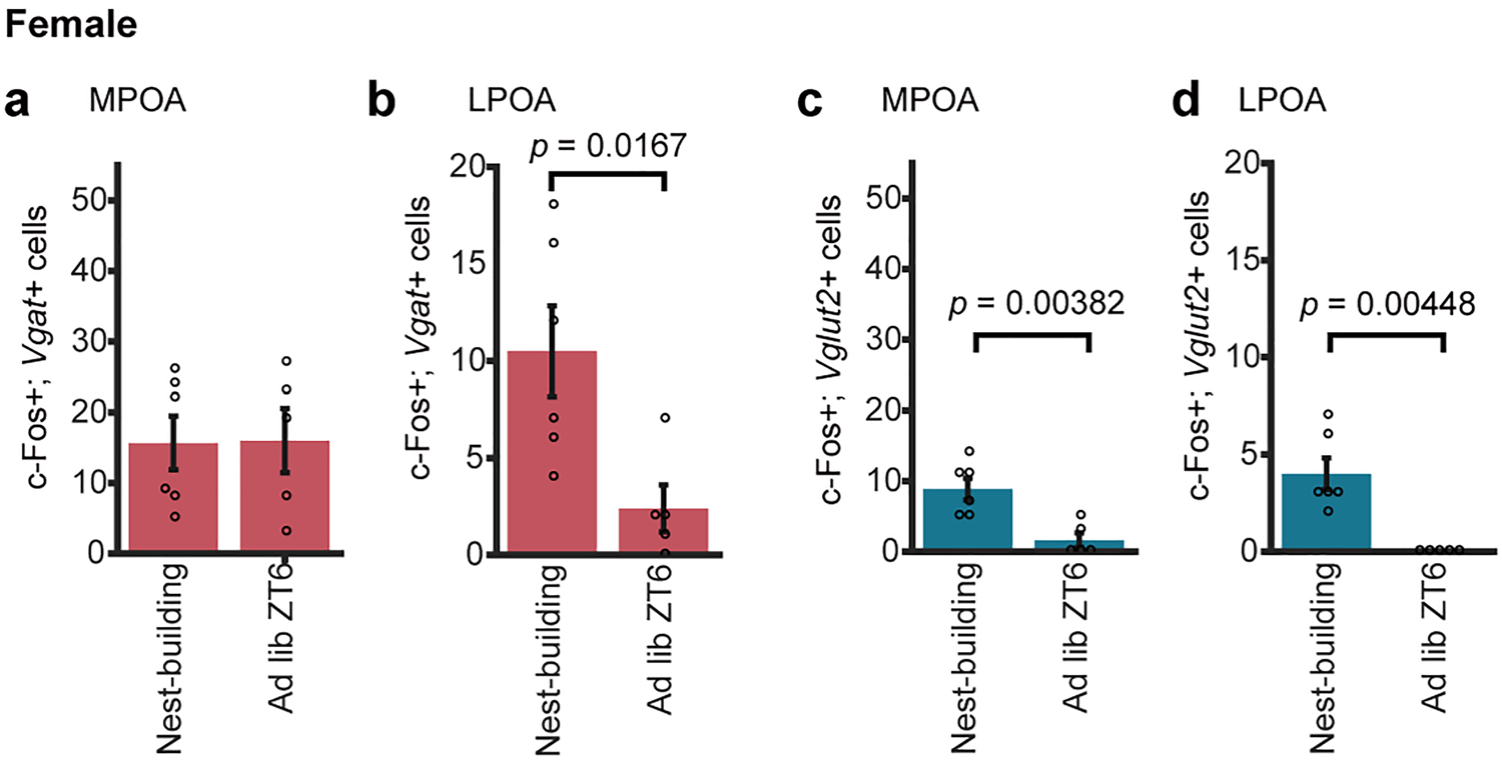
Excitatory and inhibitory populations of c-Fos-positive cells in the preoptic area associated with nest-building in females. (**a**, **b**) The number of c-Fos and *Vgat* double-positive cells in the MPOA (**a**), LPOA (**b**) in the nest-building group and ad lib ZT6 group. (**c**, **d**) The number of c-Fos and *Vglut2* double-positive cells in the MPOA (**c**) and LPOA (**d**) in the nest-building group and ad lib ZT6 group. (MPOA and LPOA: nest-building, n = 6; ad lib ZT6, n = 5). Welch's t-test. Data are shown as the mean ± s.e.m.

We also performed c-Fos immunostaining combined with double in situ hybridization for *Vgat* and *Vglut2* in females exposed to fresh nest material at ZT6 (nest-building group) and mice ad lib slept at ZT6 (ad lib ZT6 group) in the LPOA (Fig. 4a–d).

In the MPOA, the number of c-Fos- and *Vgat*-positive cells of the nest-building group was not significantly different from that of ad lib ZT6 group, whereas the number of c-Fos- and *Vglut2*-positive cells showed significant difference (nest-building vs. ad lib ZT6, p = 0.00382) (Fig. 4a,c). Among c-Fos-positive cells, the percentage of *Vgat*-positive cells among c-Fos-positive cells was greater than that of *Vglut2*-positive cells in the nest-building and ad lib ZT6 groups (Table 2).

**Table 2:** The proportion of *Vgat*-positive cells or *Vglut*-positive cells among c-Fos positive cells in each group of virgin females.

Virgin females		
	Nest-building	Ad lib ZT6
MPOA		
Vgat	59.1%	84.2%
Vglut2	33.3%	8.4%
LPOA		
Vgat	67.7%	60.0%
Vglut2	25.8%	0.0%

In the LPOA, the number of c-Fos- and *Vgat*-positive cells of the nest-building group was significantly higher than that of ad lib ZT6 group (nest-building vs. ad lib ZT6, *p* = 0.0167) and the number of c-Fos- and *Vglut2*-positive cells showed significant difference (nest-building vs. ad lib ZT6, *p* = 0.00448) (Fig. 4b,d). Among c-Fos-positive cells, the percentage of *Vgat*-positive cells among c-Fos-positive cells was greater than that of *Vglut2*-positive cells in the nest-building and ad lib ZT6 groups (Table 2). These results support that GABAergic and glutamatergic neurons in the LPOA may include neurons that are specifically activated in fresh nest material and enhance nest-building, regardless of sex.

### c-Fos-positive cells in the POA related to nest-building and parenting

**Figure 5:**
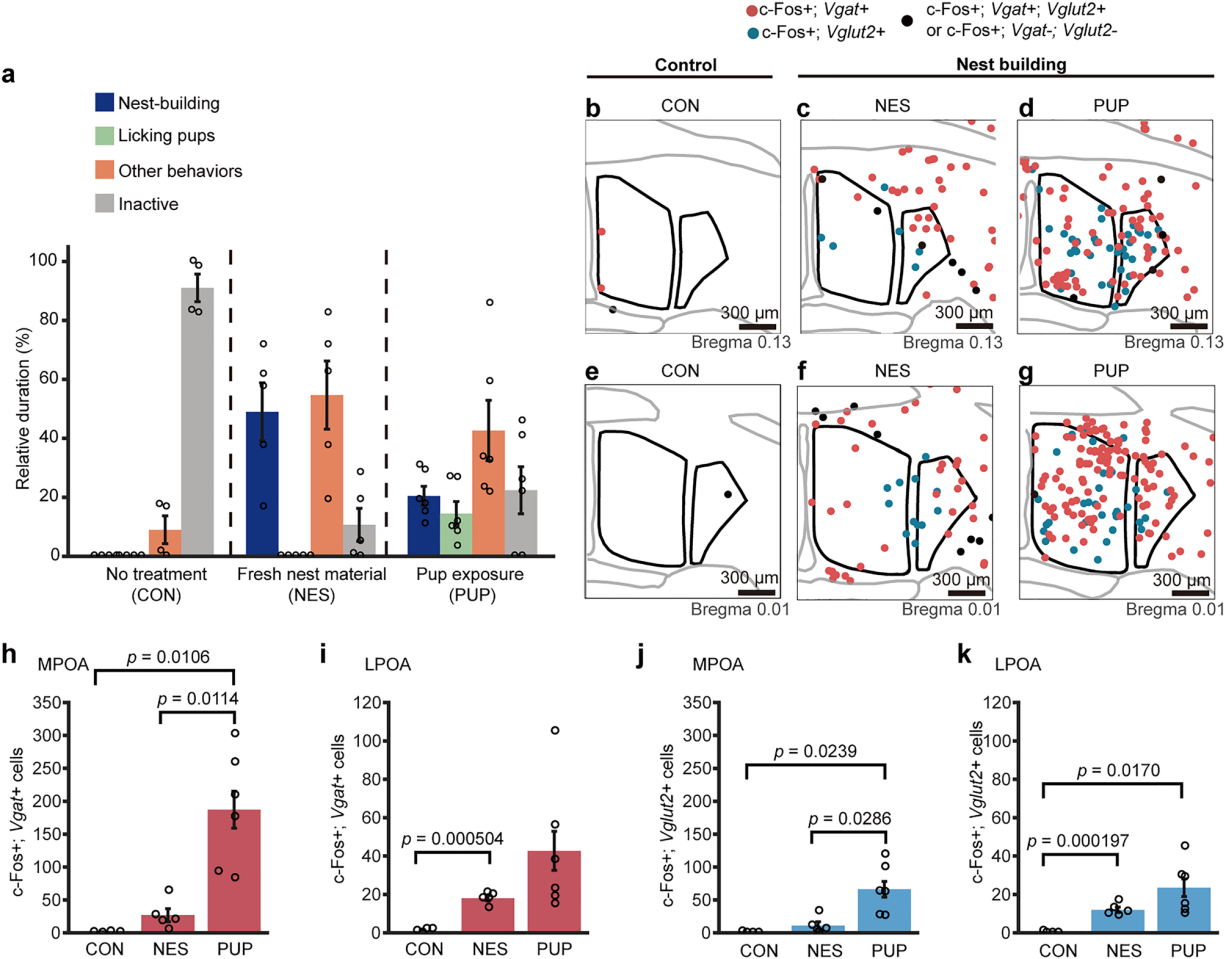
Excitatory and inhibitory populations of c-Fos positive neurons in the preoptic area associated with nest-building and parenting. (**a**) Relative duration spent in nest-building, licking pups, other behaviors and inactive during the assay for each condition. No treatment (CON), fresh nest material exposure (NES), and pup exposure (PUP). (**b**–**g**) Representative distributions of c-Fos-positive, *Vglut2*- or *Vgat*-expressing neurons for CON, NES, and PUP conditions in the MPOA and LPOA. Red circles indicate c-Fos-positive cells expressing *Vgat*. Blue circles indicate c-Fos-positive cells expressing *Vglut2*. Black circles indicate c-Fos-positive cells that express neither *Vgat* nor *Vglut2* and indicate c-Fos-positive cells that express both *Vgat* and *Vglut2*. Scale bars: 300 μm. (**h**, **i**) The number of c-Fos and *Vgat* double-positive cells in the MPOA (**h**) and LPOA (**i**) in CON, NES, and PUP groups. (**j**, **k**) The number of c-Fos and *Vglut2* double-positive cells in the MPOA (**j**) and LPOA (**k**) in CON, NES, and PUP groups. (CON, n = 4; NES, n = 5; PUP, n = 6). Each p-value was adjusted by Holm's method after Welch's t-test. Data are shown as the mean ± s.e.m.

Nest-building has been well studied as a maternal behavior in female mice and the POA has been reported to be involved in parental behavior^7,9,13^. Mating and cohabitation with female induce paternal behavior of male mice, whereas virgin male mice exhibit aggressive behavior to pups^18^. Using these sexually experienced mice after confirming their parental responsiveness, we next examined c-Fos-positive cells in response to nest-building induced by pup exposure (PUP group) and compared the number of c-Fos-positive cells to that of nest-building induced by fresh nest material (NES group). When the pups were introduced into the male cage of PUP group, all of the mice showed parental retrieving followed by nest-building and licking behaviors. Neither attack nor avoidance behavior was observed in these mice. On average, PUP group mice spent 20 ± 3% of their time building nests and 14 ± 4% on licking pups. NES group mice spend 49 ± 10% of time in nest-building (Fig. 5a). In comparison, the other male mice without any manipulation (CON group) were mainly inactive (91 ± 5% of time) Pup exposure led to a prominent increase in the number of c-Fos-positive inhibitory and excitatory cells throughout the MPOA and LPOA of males (Fig. 5b–g), which was similar to the results in females in our previous study^7^. Compared to pup exposure, the number of c-Fos-positive cells in the MPOA in response to fresh nest material or no treatment was greatly reduced in both inhibitory and excitatory neurons (inhibitory c-Fos-positive cells, PUP group vs. NES group, *p* = 0.0114, PUP group vs. CON group, *p* = 0.0106; excitatory c-Fos-positive cells, PUP group vs. NES group, *p* = 0.0286, PUP group vs. CON group, *p* = 0.0239) (Fig. 5h,j). There was no significant difference between NES and CON groups in both inhibitory and excitatory neurons. Among c-Fos-positive cells in the MPOA, the proportion of *Vgat*-positive cells was greater than that of *Vglut2*-positive cells in both NES group and PUP group (Supplementary Table S1).

In contrast, in the LPOA, the number of c-Fos-positive cells in NES group was not different from that of PUP group, in both inhibitory and excitatory neurons (Fig. 5i,k). In inhibitory neurons, the number of c-Fos-positive cells in PUP group was not significantly different from that of CON group, although the number of c-Fos-positive cells in PUP group was greater than CON group. In excitatory neurons, the number of c-Fos-positive cells in PUP group was significantly different from that of CON group (*p* = 0.0170). The number of c-Fos-positive cells in NES group was different from that of CON group, in both inhibitory and excitatory neurons (*p* = 0.000504 and *p* = 0.000197, respectively). Among c-Fos-positive cells, the proportion of *Vgat*-positive cells was greater than that of *Vglut2*-positive cells in both NES and PUP groups (Supplementary Table S1).

### Fresh nest material exposure activated *Esr1*-negative cells in the POA

**Figure 6:**
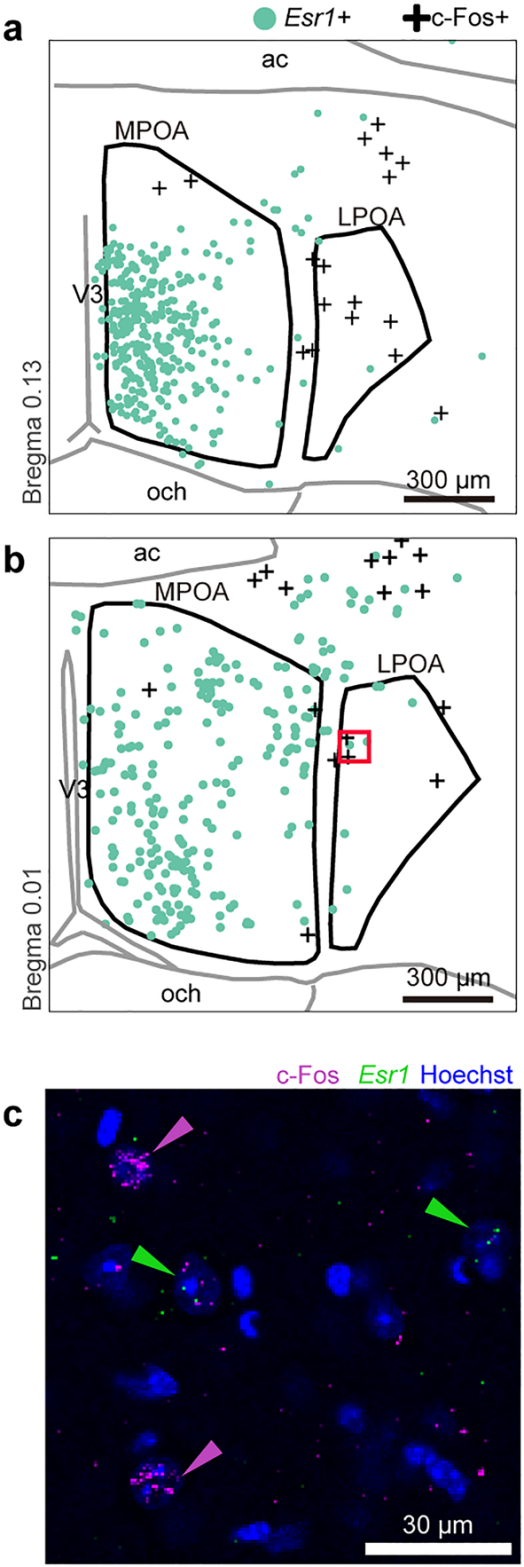
Nest-building activates Esr1-negative cells in the POA. (**a**, **b**) Distribution of *estrogen receptor 1* (*Esr1*)-positive cells and c-Fos-positive cells induced by nest-building in the POA. The red square in (**b**) corresponds to (**c**). (**c**) c-Fos-immunostaining with in situ hybridization for *Esr1* mRNA of the LPOA. Nuclei were stained with Hoechst. Scale bars; 300 μm (**a**, **b**) and 30 μm (**c**).

Given that *Esr1*-positive cells in the MPOA are involved in maternal behaviors^19^, we performed c-Fos immunostaining combined with in situ hybridization for *Esr1* mRNA in virgin male mice to examine whether neurons activated by the introduction of fresh nest material are positive for *Esr1*. *Esr1*-positive cells were abundant in the MPOA and scarce in the LPOA (Fig. 6, n = 3, MPOA, 647.0 ± 28.4 cells; LPOA, 10.7 ± 1.8 cells.), consistent with previous immunostaining for Esr1^20^. The number of c-Fos-positive cells in the MPOA was smaller than in the LPOA (Fig. 6a, *n* = 3, MPOA, 3.7 ± 0.7 cells; LPOA, 9.7 ± 1.2 cells). In virgin male mice, only a few *Esr1*-positive cells expressed c-Fos in response to nest material in the MPOA and LPOA (MPOA, 0.05% of 1941 *Esr1*-positive cells; LPOA, none of 32 *Esr1*-positive cells) (Fig. 6c).

## Discussion

The present study showed diurnal changes in spontaneous nest-building behavior of single-housed male mice and that GABAergic and glutamatergic neurons of the LPOA are activated for nest-building behaviors.

During the light phase, mice spent most of their time sleeping in high nests and were inactive, so little nest-building was observed. Similarly, around ZT20, time spent in building nests was shortest during the dark phase and most of the nests was high, when mice have “siesta” sleep^21^. In contrast, nest-building time was statistically longer at ZT23, just before the beginning of the light phase. Male mice also spent longer time in nest-building at the early to middle dark phase, but this did not reach significance due to large individual and hourly variability. The diurnal changes in spontaneous nest-building is consistent with the idea that the main aim of nest-building is preparatory to rest and sleep as recently reported^11^. In fact, inactive time ratio was greater after nest-building than before. During the early dark phase, mice not only built nests but also flattened them as the mice actively moved around and explored the cage. This is why many nests were rated as flat, even though mice spent a longer time in nest-building. It has been previously reported that when mice spent more time in nest-building, nest quality tended to be low^5^.

Although the time spent in nest-building was shorter during the light phase and at the beginning of the dark phase, the behavioral response to fresh nest material was largely different. The introduction of fresh nest material elicited robust nest-building behavior at ZT6, but little at ZT12. This result may be explained by the weaker motivation for nest-building as sleep preparatory behavior at ZT12, when sleep need is lowest during the day.

To identify the neuronal populations that are associated with nest-building behavior, we explored the brain regions that increase c-Fos positive cell number in response to fresh nest material introduction at ZT6, when spontaneous nest-building was very low. However, the introduction of fresh nest material at ZT6 in the middle of the light phase has the effect of awakening in addition to nest-building, and this awakening itself also has the effect of increasing c-Fos in various neuron groups. For this reason, we searched for sites that showed more c-Fos-positive cells for nest-building induction at ZT6 than ad lib sleep/wakefulness at ZT12, which has the highest level of spontaneous arousal. In the hypothalamus, GABAergic and glutamatergic neurons in the LPOA are the only neural group that demonstrated a significantly increased number of c-Fos-positive neurons related to nest-building behavior, compared to the naturally awake group ZT12. We also showed that GABAergic and glutamatergic LPOA neurons are activated in females after the introduction of nest material, compared to ad lib slept female group at ZT6. These results support that LPOA neurons are associated with nest-building behavior in both sexes. However, the current study does not deny the possibility that wake-response masked nest-building-specific activation of neurons. Since there are wake-promoting neurons in the LH, such as orexin-positive neurons that are active during the dark phase^22^, it may be difficult to reach a significant increase in the number of c-Fos positive cells over the ZT12 group, even if a certain population of LH neurons is activated specifically for nest-building behavior at ZT6.

In the present study, nest-building behavior induced by exposure to pups increased the number of c-Fos-positive GABAergic and glutamatergic neurons in both MPOA and LPOA. However, nest-building behavior induced by the introduction of fresh nest material increased the number of c-Fos-positive GABAergic and glutamatergic neurons in the LPOA, but not in the MPOA. These results suggest that the MPOA is involved in nest-building in relation to parental behavior, whereas the LPOA may have a population of neurons associated with nest-building in general. Consistently, chemogenetic inhibition of GABAergic neurons in the MPOA suppressed maternal nest-building^13^. Ablation of galanin neurons in the MPOA suppressed maternal nest-building^23^. Excitotoxic lesions in the central and ventral regions of the MPOA suppressed nest-building in female mice rearing pups^7^. In contrast to the MPOA, the role of the LPOA in parental nest-building has been largely unknown. However, it has been reported that pup-exposure to virgin female and father mice increased c-Fos expression in the LPOA as well as MPOA^7,24^, suggesting that the LPOA is also involved in parental behaviors. In addition, previous studies in rats have shown that the connection between MPOA and LPOA is important for maternal behavior in female rats^25^ and that lesions of the LPOA and substantia innominata impaired nest-building and other maternal behaviors in lactating females with pups^26^.

Reflecting differences in their involvement in nest-building behavior, the MPOA and LPOA are adjacent but differ in gene expression^9^. For example, galanin, neurotensin, and estrogen receptor genes are rich in the MPOA but devoid in the LPOA. The current study showed that *Esr1*-negative neurons in the LPOA and MPOA are activated in response to fresh nest materials. Consistently, optogenetic activation of *Esr1*-positive neurons in the MPOA did not enhance maternal nest-building, although optogenetic activation of *Vgat*-positive MPOA neurons enhanced maternal nest-building^13^. Pharmacological blockage of estrogen in the MPOA and knockdown of *Esr1* mRNA suppressed pup retrieval and pup licking in female mice^27,28^. These findings suggest that *Esr1*-negative LPOA neurons, *Esr1*-negative MPOA neurons and *Esr1*-positive MPOA neurons may be involved in general nest-building behavior, maternal nesting, and maternal behaviors except nest-building, respectively.

Because the purposes of nesting include sleep, rest, and temperature maintenance in addition to parental care, the different neural populations may enhance or suppress nest-building behavior in a certain context or under a specific motivation through distinct populations of MPOA neurons that are involved in various behaviors, such as body temperature regulation, sexual behaviors, and sleep^9,10,29,30^. Thus, multiple factors promote nest-building behavior, including pregnancy, low temperatures, the presence of pup and pre-sleep, and different brain regions associated with each factor likely act as regulators of nest-building behaviors^10–13^. It is unclear how the brain integrates various internal and external information to perform the same nest-building behavior, but it is likely that a common neural circuitry underlies nest-building behavior. The current results suggest the possibility that a certain population of LPOA neurons may be integrated into the neural circuitry of nest-building.

The present study has several limitations. Although we focused on the hypothalamus, other brain areas may contain neuronal groups that are involved in nest-building, such as Edinger-Westphal nucleus neurons which are necessary for preparatory nesting during pregnancy^12^. Both methods of examining nest-building behavior by introducing fresh nest material or pups inevitably result in the activation of neurons not by nest-building behavior but by accompanying behaviors and stimuli. The introduction of fresh nest material makes mice arouse and induces exploratory behaviors such as sniffing the nesting material. Removal of existing nests may cause mice to feel cold and activate cold-sensitive neurons. In the parental nest-building assay, mice not only built nests, but also retrieved pups and licked the pups. MPOA subregions are known to be differentially involved in maternal behaviors, with subregion-specific c-Fos expression^31–35^. Thus, future manipulative studies are necessary to investigate the causal role of GABAergic and glutamatergic LPOA neurons in nest-building not only as a rest and sleep preparatory behavior as well as parental behavior.

## Methods

### Animals

All procedures were carried out in accordance with ARRIVE guidelines and the Guidelines for Animal Experiments of Toho University and were approved by Toho University Animal Care and User Committee (#21-53-405). Male and female C57BL/6J mice were obtained from Japan SLC Inc. (Shizuoka, Japan) and CLEA Japan, Inc. (Tokyo, Japan). Mice were bred and maintained in our colony with controlled temperature (23 ± 2 °C) and humidity (50 ± 10%), ad libitum access to water and food, and under 12 h light–dark cycle (ZT0 = lights on and ZT12 = lights off). Mice were group housed by sex after weaning at 4 weeks of age. Prior to behavioral experiments, mice were individually housed in shoebox cages (27 cm × 18 cm × 13 cm) with aspen chips (CLEA Japan, Inc., Tokyo, Japan) and a piece of cotton square (5 cm × 5 cm, Nestlet, Animec) as nest material. Mice aged 8–18 weeks were used for all studies. All mice were used after at least 2 days single housing. All female mice had no previous reproductive experience. The estrus cycle state was not checked. Father mice were co-housed with one or two female mice until the late pregnancy of either female mouse^8^, and then individually housed and provided with nest material as described above. Paternal behavior of father mice was confirmed by the introduction of pups after at least two days of single-housing.

### Assessment of nest-building behavior

Home cage behaviors of male mice were video-recorded with the infrared-sensitive camera from the side to quantify the duration of nest-building behavior of virgin male mice. Video-recording during the dark phase was performed under infrared illuminance. The mice were housed at least for a month in the testing room for video recording. The mice were recorded after 5 days or more from the introduction of the nest material. The mice cages were moved to the video-recording space in the same room more than an hour before starting video-recording. We classified mouse behaviors into the following categories on a minute-by-minute basis: (1) Nest-building: This includes gathering, reshaping, and pushing nest material and burrowing into the nest, (2) Active: This category includes rearing, travelling, cage-lid hanging, or any head movement, excluding next-building behaviors, (3) Inactive: a mouse is not traveling and had no head movement for 20 s; (4) Invisible: a behaving mouse is completely covered by the nest, making it impossible to classify mouse behavior into nest-building or active. Alternatively, the mouse is obscured by shadows and its behavioral category as inactive or active cannot be determined. In addition, nest height was rated on a three-point scale of “flat”, “low” and “high”. Nests were judged as flat when there was no noticeable nest because nest material was scattered or flattened. Nests were judged low when the nest was recognizable, but the nest height was lower than half the height of the mouse body. Nests were judged high when the nests were taller than half the height of the mouse body. The relative duration of nest-building and nest height was quantified by an hour. Behavior of each mouse was continuously recorded for 10–24 h (3 mice were recorded for 17 h from ZT10 to ZT3, 2 mice were recorded for 10 h from ZT2 to ZT12 and 3 mice were recorded for 24 h). Due to animal facility and equipment constrains, the room for recording could not be occupied for more than 24 h, and 3 mice were recorded for 17 h from ZT10 to ZT3 and 2 mice were recorded for 10 h from ZT2. These periods were chosen so that these data sets covered 24 h of recording. The time points categorized as “Inactive” for more than 30 min in an hour were excluded from the calculation for the relative duration of nest-building. 4–8 mice were consequently observed for each period (ZT10-12: 8 mice; ZT12-20: 6 mice; ZT20-22: 5 mice; ZT22-2: 6 mice; ZT2-3: 8 mice; ZT3-5: 4 mice; ZT5-9: 5 mice).

### Nest-building assay

Five days or more after mice were provided with nest material, nests were observed to have been built in the cage. To perform nest-building assay, build-established nests in the cage were gently removed, and then fresh nest material was introduced to the cage at ZT6 or ZT12 for male mice and at ZT6 for female mice. The behavior of mice was observed by an infrared-sensitive camera as needed. The behaviors were assessed every minute and categorized as nest-building (handling or biting nest material) or active (excluding nest-building) or inactive.

### Animals for histological study

For the nest-building group, 120 min after the removal of the established nest and the introduction of fresh nest material at ZT6, the mice were deeply anesthetized with sodium pentobarbital (50 mg/kg, i.p.) and proceeded to histological examination. In general, c-Fos protein begins to be expressed 60 min after stimulation, the expression level reached to maximum at 2–4 h, and then gradually decreases with 2 h half-life, but the actual peak depends on the stimuli^36–38^. Previous studies examined c-Fos expression 120 min or longer after parental and nest-building behaviors^7,11^.

The mice which successfully performed nest-building were used for histological analyses. For assessing c-Fos expression in the active and inactive state, the individually housed mice were euthanized with sodium pentobarbital (50 mg/kg, i.p.) at ZT14 and ZT8 without any manipulation, respectively. These histological sampling were performed on both sexes except examination of active state. For the examination of active state, only male mice were used.

To examine the c-Fos expression induced by parental nest-building, more than two days after confirming the parental responsiveness of father mice, foreign three pups (1–7 days old) were introduced in the father mice cage at the early light phase (ZT2-ZT6). Father mice were corded their behavior every 15 s for 30 min. We then calculated the relative duration as the proportion of time spent in nest-building behavior, licking pups in the nest, other behaviors (e.g. feeding and walking), and inactivity. Then, the father mice were sacrificed 120 min after the pup introduction. The mice that performed nest-building during the first 30 min from the introduction of the pups were used for assessing c-Fos expression. The brain of father mice were also sampled in conditions without pups. To examine the c-Fos expression induced by nest-building, 120 min after the nest-building assay at ZT6, the father mice were sacrificed. For the no treatment control, the singly-housed father mice were also sacrificed at ZT8.

### Preparation of brain sections for ISH combined with IHC

Mice were deeply anesthetized with sodium pentobarbital (50 mg/kg, i.p.) and then perfused transcardially with 4% (w/v) paraformaldehyde (PFA) in phosphate-buffered saline (PBS). The brains were removed and post-fixed in the same fixative and stored at 4 °C overnight, followed by cryoprotection of 30% (w/v) sucrose in PBS for 2 days, embedded in FSC22 Frozen Section Compound (Leica, Wetzlar, Germany) and stored at – 80 °C until cryosectioning. Brains were cryosectioned coronally at a thickness of 40 µm. Every third section from the serial sections was processed for ISH combined with IHC.

### ISH combined with IHC

ISH combined with IHC was performed by ISH HCR using short hairpin DNAs as previously described^39,40^ with some modifications. All procedures were performed on free-floating sections. The sections were washed with PBS containing 0.2% Triton (PBST) two times for 5 min, immersed in methanol for 10 min, and washed with PBST two times for 5 min. After washing, the sections were prehybridized for 10 min at 37 °C in hybridization buffer containing 30% formamide, 10% dextran sulfate, 5 × saline sodium-citrate buffer (SSC), 0.1% Tween20, 50 ug/ml heparin, and 1 × Denhardt’s solution. The sections were treated with another hybridization solution containing a mixture of 20 nM probes for *Vgat* and *Vglut2* mRNAs or a mixture of 20 nM probes for *Esr1* mRNAs, and these probes contained the split-initiator sequences for amplification of S41, S45, and S86 hairpin DNAs (Supplementary Table S2), respectively. Probes were denatured for 90 s at 95 °C before use. The sections were incubated in the hybridization solution overnight at 37 °C. For negative controls, the sections were treated with a hybridization solution without probes. We confirmed no meaningful signals without probes. After hybridization, the sections were washed three times for 10 min in 5 × SSC with 0.1% Tween20 (SSCT) and 30% formamide at 37 °C, followed by three washes for 10 min in 5 × SSCT without formamide at 37 °C. For quenching autofluorescence, the sections were bleached by the LED illuminator (TiYO, Nepagene, Japan) for 30 min in 5 × SSCT. For HCR amplification, either one or two of SaraFluor488-conjugated S45, ATTO550-conjugated S41, and SaraFluor488-conjugated S86 hairpin DNA pairs were used (Supplementary Table S3). These hairpin solutions were separately snap-cooled before use. The sections were incubated in an amplification buffer (10% dextran sulfate in 8 × SSC, 0.2% TritonX-100, 100 mM MgCl_2_) for 5 min at 25 °C, and HCR amplification was performed in another amplification buffer containing 60 nM hairpin DNAs for 2 h at 25 °C. The sections were washed three times for 10 min in 5 × SSCT at room temperature, followed by PBST washing for 5 min. The sections were blocked using 0.8% BlockAce (Dainihon-Seiyaku, Japan) in PBST for 30 min, which was followed by overnight incubation with rabbit anti-c-Fos antibody (1:4000, sc-52, Santa Cruz) in 0.4% BlockAce/PBST at 4 °C. The sections were washed three times for 10 min in PBST at room temperature and incubated with a 647 anti-rabbit antibody (1:1000, A31573, Invitrogen) with Hoechst 33342 (1 µg/ml) for an hour at room temperature. The sections were washed two times for 5 min in PBST and 5 min in PBS. The sections were mounted with an anti-fade reagent (VECTASHIELD Mounting Medium, Vector Laboratories).

### Histological analysis

Photomicrographs were obtained by a Nikon Eclipse Ni microscope equipped with A1R confocal detection system (Nikon Instruments Inc., Tokyo, Japan) under 20 ×/0.75 NA objective lenses. The images were analyzed using ImageJ (NIH, USA). The borders of brain regions were identified based on the brain atlas^41^ except preoptic area, and the border of medial and lateral preoptic areas was identified based on our previous studies^7,8,20^. The contours for each brain region were set inside the border of the brain region as shown in Fig. 2.

To increase the reproducibility of selecting contours, the location of contours was adjusted based on the *Vgat*/*Vglut2* mRNA distribution rather than the relative location because all sections were not identically prepared between samples in their tilt. The c-Fos-immunoreactive cells were bilaterally selected on the threshold images using Analyze particle function of ImageJ software (ver. 1.53q, NIH). The threshold was determined to be above background or nonspecific signals on the control sections, and the same threshold was used through each analysis. There were the data that lost a lateral section. The *Vgat*, *Vglut2*, or *Esr1* positive cells were manually marked on the threshold images when the granule-like signals were present close to the cell nuclei. The number of c-Fos-positive cells was calculated in the contours for each brain regions in Fig. 2, bregma 0.13 mm and 0.01 mm for the MPOA and the LPOA in Figs. 3, 4, 5, and 6, in 300 µm squares on the section bregma − 0.59 mm and − 0.71 mm for the AH, in 400 µm squares on the sections bregma − 1.31 mm and − 1.43 mm for the DMH, in 300 µm × 600 µm squares on the sections bregma − 1.31 mm and − 1.43 mm for the LH, and in 300 µm squares on the sections bregma − 2.15 mm and − 2.27 mm for the PH in Fig. 3. All procedures were performed under blind conditions.

### Statistical analysis

We analyzed the diurnal variation in Fig. 1f by general liner model (GLM). In the GLM, individual mice and time points were used as factorial variables to deal with repeated measured values of the time spent in building nests. The statistical significance of the factors was tested by Type II tests. The significance of each time point was analyzed by Wald test. We used paired t-test for the comparison of accumulated nest-building time in “ZT6 Male” and “ZT12 Male” groups (paired t-test) in Fig. 1h and the comparison of inactive time in “pre nest-building” and “post nest-building”. We used Welch’s t-test for all the number of c-Fos cell comparisons. Welch’s t-test was used without assuming normality of the data because Welch’s t-test is robust for samples with high skewness values even when the test is applied without establishing normality^42–44^ Data were shown as Mean ± SEM. *p* values of all multiple comparison tests were adjusted by Holm’s method. The GLM was performed using R 4.2.1. Statistical analyses except for the GLM were performed using the statistical package statsmodels in Python^45^.

### Supplementary Information

Supplementary Information 1.

Supplementary Video 1.

## Data Availability

All data and Python scripts used in this study are available from the corresponding authors without restriction.
