# Supplementary material for: Activation of lateral preoptic neurons is associated with nest-building in male mice
Source: Sci Rep. 2024 Apr 9;14:8346. doi: 10.1038/s41598-024-59061-z (PMC11004109; doi:10.1038/s41598-024-59061-z)
Supplement: Supplementary file 1 — Supplementary Information 1. [file 41598_2024_59061_MOESM1_ESM.pdf]

Supplementary Information

Activation of lateral preoptic neurons is associated with nest-building in male mice

Natsuki Tagawa1,2, Keita Mori2, Michinori Koebis3, Atsu Aiba2,3, Yuichi Iino2, Yousuke Tsuneoka1*, Hiromasa Funato1,4*

[1]Department of Anatomy, Graduate School of Medicine, Toho University, Tokyo 143-8540, Japan. [2]Department of Biological Sciences, Graduate School of Science, The University of Tokyo, Bunkyo-ku, Tokyo, 113-0033, Japan. [3]Laboratory of Animal Resources, Center for Disease Biology and Integrative Medicine, Graduate School of Medicine, The University of Tokyo, Tokyo 113-0033, Japan. [4]International Institute for Integrative Sleep Medicine (IIIS), University of Tsukuba, Tsukuba, Japan

* Correspondence:
Yousuke Tsuneoka, yousuke.tsuneoka@med.toho-u.ac.jp
Hiromasa Funato, hiromasa.funato@med.toho-u.ac.jp

Supplementary Table S1. The proportion of *Vgat*-positive cells or *Vglut*-positive cells among c-Fos positive cells in each group of fathers.

		NES	PUP
**MPOA**	Vgat	65.9%	70.7%
	Vglut2	26.9%	25.0%
**LPOA**	Vgat	59.5%	61.7%
	Vglut2	35.4%	34.0%

Supplementary Table S2. Sequences of probe sets

The nucleotide sequences of probe sets that were used for in situ hybridization chain reaction to detect mRNA are shown.

Probe name	First probe	Second probe
Vgat-1S41	GCTCGACGTAACCACGACGTA CAAGATACACGTCAT	AACTGTTGTACATGAGGTTGCCG CTAATCCTTTGCAACA
Vgat-2S41	GCTCGACGTAACTTGAGATTC TTCAGGAAGGCGCAG	ACACAGCAGACTGAACTTGGAC ACGAATCCTTTGCAACA
Vgat-3S41	GCTCGACGTAACACCTTCTCC CAGGCCCAATCACGC	AAACTTCTTGACGTCGATGTAGA ACAATCCTTTGCAACA
Vgat-4S41	GCTCGACGTAACTGCTGCATG TTGCCTTCGAGAGAG	GTTCATCATGCAGTGGAATTCGC TGAATCCTTTGCAACA
Vgat-5S41	GCTCGACGTAAGAGAGACTTC TCCAGCACTTCGACG	GAAGAAGGCGCGACTGCCTTCC TGGAATCCTTTGCAACA
Vgat-6S41	GCTCGACGTAAATGGCCATGA GCAGCGTGAAGACCA	ATGAGCAGCGCGAAGTGTGGCA CGTAATCCTTTGCAACA
Vgat-7S41	GCTCGACGTAATGCCACAGCA GCTTGCGCCAGAGAA	AAGATGGCCACATCGAAGAAGA CCTAATCCTTTGCAACA
Vgat-8S41	GCTCGACGTAATGATGAGGAA CAACCCCAGGTAGCC	TGTAGCAGCACACCACTGCGGC GAAAATCCTTTGCAACA
Vgat-9S41	GCTCGACGTAATATGGCCACAT ACGAGTCCCGCACG	GAATCGAGGAGCGCAGCATGCG TTAAATCCTTTGCAACA
Vgat-10S41	GCTCGACGTAACCTCGATGAG ACCCTCGAGTGAATG	CCTAGTCCTCTGCGTTGGTTCGG TAAATCCTTTGCAACA
Vglut2-1S45	CCTCCACGTAACACACAGCTG TGGTTTAGGCAGCGC	TCTTTGCGAACGTGAGTGAATAA CAAATCCATCTAAGCT
Vglut2-2S45	CCTCCACGTAATCTCCTCCCAT CGCGCCGGCGTAAT	TGCTGTTAGCTTCCTGCCTTAAC ATAATCCATCTAAGCT
Vglut2-3S45	CCTCCACGTAATGTTCTGGAA GTCACCTCACCGAAA	GTATTTGGGCGGGTGCTGATGAG TCAATCCATCTAAGCT
Vglut2-4S45	CCTCCACGTAACTAATAGGAG AATTGGTACACACAG	AATAGCTGCATGCAGCCCACGGG TTAATCCATCTAAGCT
Vglut2-5S45	CCTCCACGTAATGGCTATGAA	TCCAGCCTTACCAGATTTAAATT

	AGACGGATTCTGCGC	GTAATCCATCTAAGCT
Vglut2-6S45	CCTCCACGTAACACCCTGTAG ATCTGTCCGAGGGAT	CTCTCGGTTGTCCTGCTTCTTCTC CAATCCATCTAAGCT
Vglut2-7S45	CCTCCACGTAAGTAAGATTTG GTGGTACCGTAATTT	GCCTCCATTCTCCTGTGAGGTAG CAAATCCATCTAAGCT
Vglut2-8S45	CCTCCACGTAAATGGGAATCT CATGGTCTGTTTTGA	CGTGACAACTGCCACAGATTGC ACTAATCCATCTAAGCT
Vglut2-9S45	CCTCCACGTAAGTGCCAACTG TTGTAGTTGTGCTCT	GGCTTCCTAACTTCTGTAGGATG ACAATCCATCTAAGCT
Vglut2-10S45	CCTCCACGTAAGTCTCTAGAC AGGCTTTCCCTGGGA	ATCTAGCCATCTTTCCTGTTCCAC TAATCCATCTAAGCT
Vglut2-11S45	CCTCCACGTAAACCCAAAACT GACAGGTAAACACAC	GAGCACAGGACACCAGACAGAT CAAAATCCATCTAAGCT
Vglut2-12S45	CCTCCACGTAATTCTTAAGGTC AGGAGTGGTTTGCA	AGACACAAAGCAGAGAGGGACT TCAAATCCATCTAAGCT
Vglut2-13S45	CCTCCACGTAAAAATCCTTTGT TTTACCGACTCCAT	TCTTTATCCCCTCTTTCCCCGGGG CAATCCATCTAAGCT
Vglut2-14S45	CCTCCACGTAACATATTGAGG GTAGAGGTGAGCAGT	ATGCACTCTGGCTGCAGATGGGA TCAATCCATCTAAGCT
Vglut2-15S45	CCTCCACGTAAACATGTACCA GACCATGCCAAAGCT	GGCTCTCATAAGACACCAGAAG CCAAATCCATCTAAGCT
Vglut2-16S45	CCTCCACGTAATGCTCTCCTCT ATGTACCTACGTTC	CACCTAGCAGATTTGCGCTCTCT CCAATCCATCTAAGCT
Vglut2-17S45	CCTCCACGTAATGGCCACCCC TCTAGTATGAGAGTA	ATCCTACTGCAAGCACCAAGAA GGAAATCCATCTAAGCT
Vglut2-18S45	CCTCCACGTAAATACTGCCATT CTTCACGGGACTTA	GTGGACGAGTGCAGCAATGAGG AAGAATCCATCTAAGCT
Esr1-1S86	GCTGGTCGGAAAATCCGTTCC GAGGCTGTTATGACC	GGAGCCTTGTGTTTTCGGTTTTG GTAATGGATTGAGTGT
Esr1-2S86	GCTGGTCGGAATCATGGTCAT GGTAAGTGGCAGCCG	CCATTCCCGAGGCTTTGGTGTGA AGAATGGATTGAGTGT
Esr1-3S86	GCTGGTCGGAATTACTGCTGG ACAGTCTCTCTCGGC	GACTCCATGATCATGTTTCCTTTC TAATGGATTGAGTGT
Esr1-4S86	GCTGGTCGGAAGGCACAGTAG CGAGTCTCCTTGGCA	GTAGCCAGAGGCATAGTCATTGC ACAATGGATTGAGTGT

Esr1-5S86	GCTGGTCGGAATGCTTCAACA TTCTCCCTCCTCGGC	CCTTCCAAGTCATCTCTCTGACG CTAATGGATTGAGTGT
Esr1-6S86	GCTGGTCGGAATCCTGAAGCA CCCATTTCATTTCGG	TGGCCAAAGGTTGGCAGCCCTC ATGAATGGATTGAGTGT
Esr1-7S86	GCTGGTCGGAATAGTGTGCTT AATCACAAGAGGGCT	TCAAGGACAAGGCAGGGCTATT CTTAATGGATTGAGTGT
Esr1-8S86	GCTGGTCGGAATTCACTGAAG GGTCTAGAAGGATCA	GTTGGTCAATAAGCCCATCATTG AGAATGGATTGAGTGT
Esr1-9S86	GCTGGTCGGAATATGAACCAG CTCCCTATCTGCTAG	CTGGCACTCTCTTTGCCCAGTTG ATAATGGATTGAGTGT
Esr1-10S86	GCTGGTCGGAATCATGGAGAT TCAAGTCCCCAAAGC	CAGGCACACTCGAGAAGGTGGA CCTAATGGATTGAGTGT
Esr1-11S86	GCTGGTCGGAAGTTAGGAGCA AACAGGAGCTTCCCC	TTTACCTTGATTCCTGTCCAGGA GCAATGGATTGAGTGT
Esr1-12S86	GCTGGTCGGAACCCTGCAGGT TCATCATGCGGAACC	ATGGATTTGAGGCACACAAACTC TTAATGGATTGAGTGT
Esr1-13S86	GCTGGTCGGAACGTGTACACT CCGGAATTAAGCAAA	CAGAGACTTCAAGGTGCTGGAC AGAAATGGATTGAGTGT
Esr1-14S86	GCTGGTCGGAACACGGTGGAT GTGGTCCTTCTCTTC	TCAAAGTGTCTGTGATCTTGTCC AGAATGGATTGAGTGT
Esr1-15S86	GCTGGTCGGAAGTGGTGGCCA GCTGGGTCTGGCTGG	TGTAAGGAATGTGCTGAAGTGG AGCAATGGATTGAGTGT
Esr1-16S86	GCTGGTCGGAATGCTAGTCAT ACATGACATGGGTAA	CCACAGTGTACGCAGGAGACAG AATAATGGATTGAGTGT
Esr1-17S86	GCTGGTCGGAAGAAAGCCACT GGTGGTGGATGCATG	CAAGCAAATAAATGGCCACTCAT ACAATGGATTGAGTGT
Esr1-18S86	GCTGGTCGGAAAGAAGACATG CCACTAAGAACTGAG	CTGGAATCCCTTTTGCCTGTTCC CAAATGGATTGAGTGT
Esr1-19S86	GCTGGTCGGAAGAAGTGCTTA TCAGAGGTGAAATGT	GCTGAGGCTTCCTCTTGGAGCCA TTAATGGATTGAGTGT
Esr1-20S86	GCTGGTCGGAACTCAAGCTGC CTTTACTGCAAGCCC	AGAATGTCCAGTTTACCAGAACA GGAATGGATTGAGTGT

Supplementary Table S3. Hairpin sequences and conjugated fluorophores

The nucleotide sequence of the hairpin DNA used for in situ hybridization chain reaction to detect mRNA and the fluorophores bound to the hairpin DNA are shown.

Hairpin ID		Sequence	Fluorophore
S41	H1	TGTTGCAAAGGAACGTCGAGCTGTAATG GTGCTCGACGTTCC	ATTO550
	H2	GCTCGACGTTCCTTTGCAACAGGAACGT CGAGCACCATTACA	
S45	H1	AGCTTAGATGGAACGTGGAGGTAACAAG CACCTCCACGTTCC	SaraFluor488
	H2	CCTCCACGTTCCATCTAAGCTGGAACGTG GAGGTGCTTGTTA	
S86	H1	ACACTCAATCCACCGACCAGCACAAGTC ATGCTGGTCGGTGG	SaraFluor488
	H2	GCTGGTCGGTGGATTGAGTGTCCACCGA CCAGCATGACTTGT	

Supplementary Figure S1. Individual nest height scales were evaluated at that point every hour. A block indicates individual nest height at that hour. Each row indicates data from a mouse.

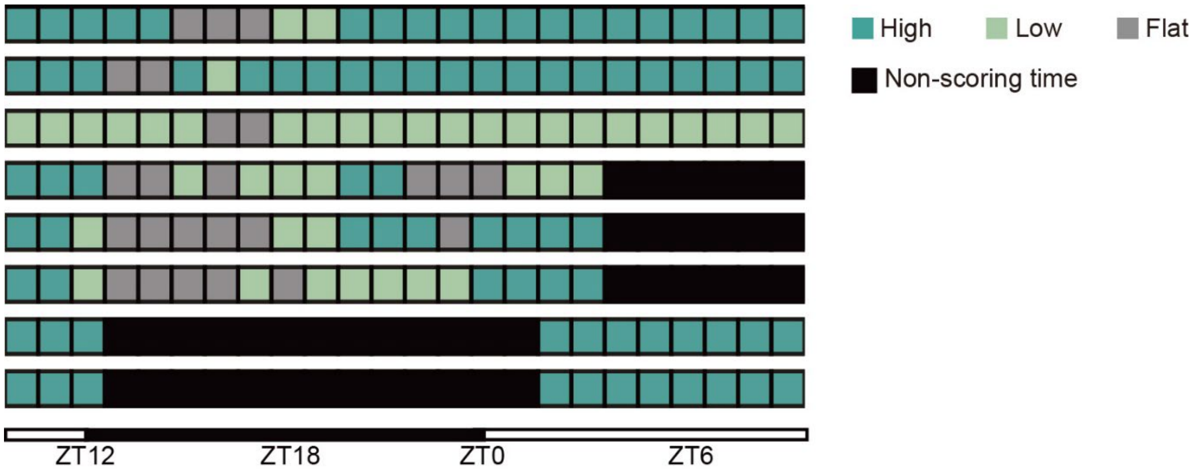

Supplementary Figure S2. The comparison of the behavioral pattern 30 minutes before and after nest-building behavior.

(a) Event plots for 30 minutes before and after continuous nest-building behavior. One plot indicates a behavior categorized per minute. A blue plot indicates "nest-building," an orange plot indicates "active", a gray plot indicates "inactive" and a black plot indicates "invisible". (b-c) The comparison of inactive time 30 minutes before and after inactive time for 30 min prior to nest-building behavior in the dark phase (b) and the light phase (c). Paired t-test.

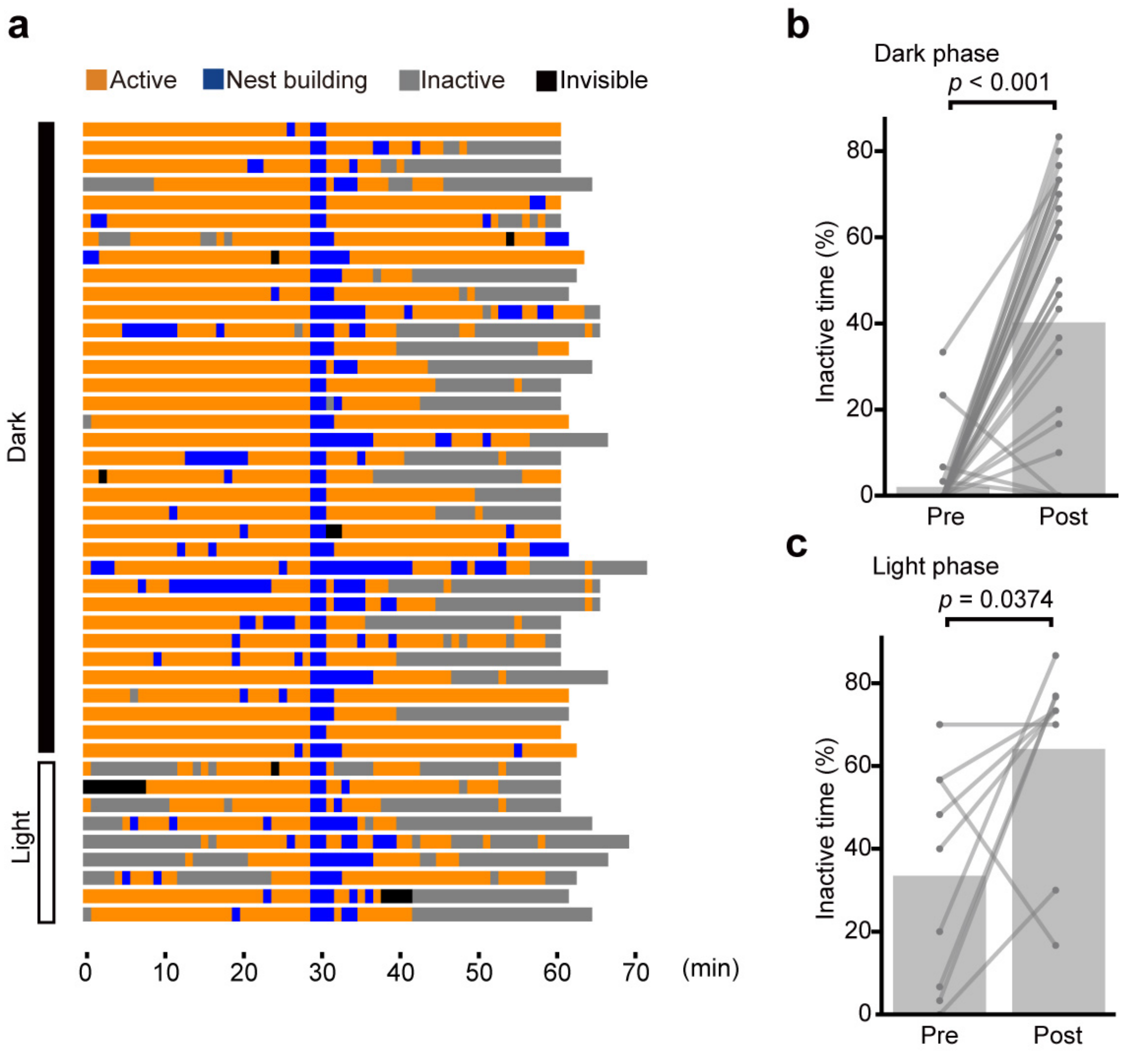

Supplementary Figure S3. Event plots for 90 minutes following the introduction of fresh nest material.

Each row indicates data from a mouse.

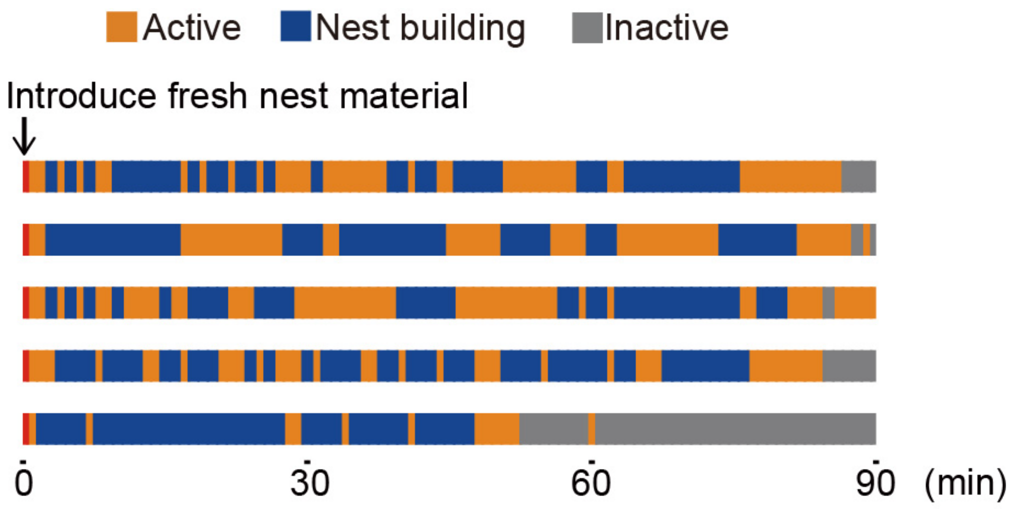

Supplementary Figure S4. No correlation of the age of the mice in nest-building group and the number of c-Fos positive cells in the POA in Figure. 3

(a-d) The correlation of the age of the mice in nest-building group and the number of c-Fos and Vgat positive cells in the MPOA (a), the number of c-Fos and Vgat positive cells in the LPOA (b), the number of c-Fos and Vglut2 positive cells in the MPOA (c), and the number of c-Fos and Vglut2 positive cells in the LPOA (d). Two-sided Spearman correlation coefficient.

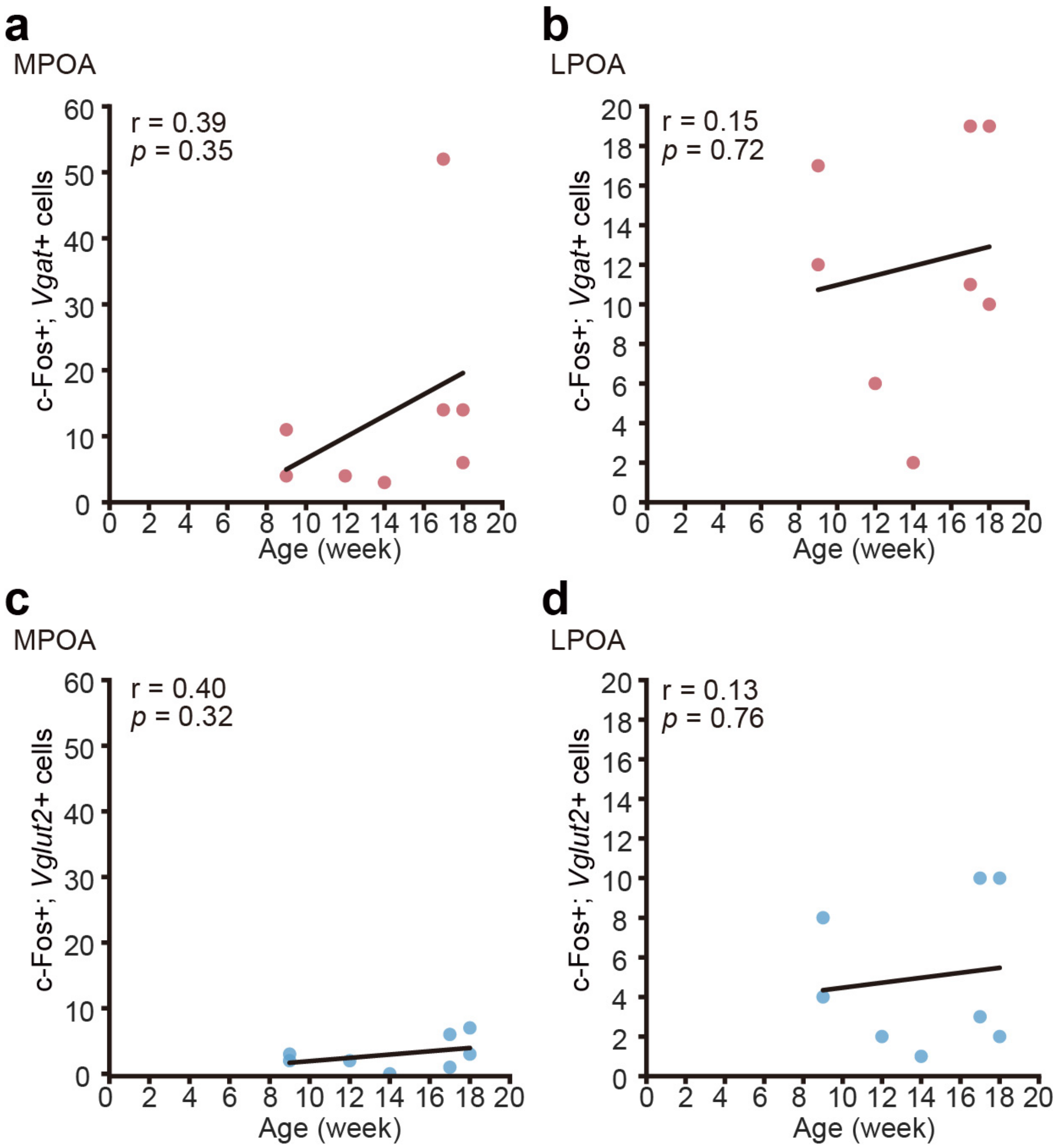

Supplemental video. Nesting-related behaviors.

When mice build nests, mice exhibit a variety of behavioral elements such as gathering, reshaping, pushing nest material, and burrowing into the nest. Related to Figure1.
